# Development of the Mechanisms Underlying Audiovisual Speech Perception Benefit

**DOI:** 10.3390/brainsci11010049

**Published:** 2021-01-05

**Authors:** Kaylah Lalonde, Lynne A. Werner

**Affiliations:** 1 Center for Hearing Research, Boys Town National Research Hospital, Omaha, NE 68131, USA; 2 Department of Speech and Hearing Sciences, University of Washington, Seattle, WA 98105, USA; lawerner@uw.edu

**Keywords:** audiovisual, multimodal cues, speech perception, development, infants, children

## Abstract

The natural environments in which infants and children learn speech and language are noisy and multimodal. Adults rely on the multimodal nature of speech to compensate for noisy environments during speech communication. Multiple mechanisms underlie mature audiovisual benefit to speech perception, including reduced uncertainty as to when auditory speech will occur, use of correlations between the amplitude envelope of auditory and visual signals in fluent speech, and use of visual phonetic knowledge for lexical access. This paper reviews evidence regarding infants’ and children’s use of temporal and phonetic mechanisms in audiovisual speech perception benefit. The ability to use temporal cues for audiovisual speech perception benefit emerges in infancy. Although infants are sensitive to the correspondence between auditory and visual phonetic cues, the ability to use this correspondence for audiovisual benefit may not emerge until age four. A more cohesive account of the development of audiovisual speech perception may follow from a more thorough understanding of the development of sensitivity to and use of various temporal and phonetic cues.

## 1. Introduction

Studies of the development of speech perception in infancy and childhood have provided valuable information about the structure of phonological representations and the mechanisms of early speech and language learning [1,2]. However, relatively few studies have considered the multimodal nature of speech perception and the noisy environments in which infants and children learn speech and language. Adults rely on the multimodal nature of speech perception to compensate for noisy environments. This article reviews evidence regarding infants’ and children’s use of the mechanisms that underlie adults’ audiovisual speech perception benefit, with a particular focus on studies that differentiate between the use of temporal and phonetic cues. The review highlights how developmental constraints on test methodology limit the ability to compare across age groups and to differentiate between sensitivity to cross-modal associations and their use for audiovisual benefit. The current state of the literature suggests that the ability to use temporal cues for audiovisual speech perception benefit emerges in infancy, whereas the ability to use correspondences between auditory and visual phonetic cues may not emerge until 4 years of age. Moreover, simpler, more salient cues can be used earlier in development. We suggest that a more thorough characterization of development of the use of various temporal and phonetic cues will result in a more cohesive account of audiovisual speech perception development.

## 2. Our Natural Environments Are Noisy and Multimodal

The natural environments in which infants and children learn speech and language are specified by highly redundant and synchronous multimodal signals. These environments are also noisy [3,4,5,6,7]. Infants are bombarded with large amounts of sensory information. They rely on the spatial and temporal coincidence of sensory information across modalities (“amodal” cues) to parse sensory input into events and objects [8,9]. Infant dishabituation responses and neural responses are stronger for synchronous audiovisual cues than for their isolated auditory and visual components [10,11,12,13,14,15,16,17,18]. They also show preference for synchronous audiovisual speech over asynchronous audiovisual speech [19].

## 3. Adults Rely on the Multimodal Nature of Speech to Compensate for Noisy Environments

Speech is a particularly rich multimodal signal. Visible articulations (and even head movements) correlate with multiple cues in the auditory speech stream, including onsets and offsets, amplitude modulations, and rhythm [20,21,22,23]. The redundant, multimodal nature of speech is advantageous. Numerous studies have demonstrated that adults rely on the multimodal nature of speech to compensate for noisy environments [24,25]. Adults detect, discriminate, and recognize speech in noise better when viewing a talker’s face than when they do not have access to visual cues [26,27,28,29,30]. For example, viewing a talker’s face while hearing speech allows adults to recognize words in noise with up to 45% greater accuracy than when listening without visual cues [28]. Viewing the talker’s face also decreases the effort adults expend when listening to speech in noise [31,32,33].

## 4. A Coherent Account of Audiovisual Speech Perception Development Has Yet to Emerge

Development of the ability to use visual speech cues to understand speech in noise extends into adolescence, with young children showing limited benefit, e.g., [34,35]. This protracted developmental time course is seemingly at odds with the fact that infants have very early awareness of the common properties of visual and acoustic speech. Infants preferentially look at a face that matches the vowel they are hearing over the same simultaneously presented face articulating a different vowel [36,37,38,39,40,41]. In 5- to 15-month-olds, this preference extends to disyllabic [42] and trisyllabic nonwords [43]. Even newborns can match auditory sentences to point-line displays of faces [44].

One interpretation of these findings is that development follows a u-shaped trajectory, wherein infants are sensitive to the correspondence between auditory and visual cues, but children are not. Another interpretation is that different studies and methods have different cognitive requirements. Infant matching studies only measure whether infants *are sensitive to the correspondence* between auditory and visual speech cues, whereas child and adult studies measure whether children and adults *use the correspondence to benefit* from visual speech cues. As Shaw and Bortfeld [45] eloquently pointed out, there is an important distinction between associating auditory–visual cues and integrating them, and techniques compatible with testing infants typically cannot differentiate these two processes. Differences in the methods used to test audiovisual speech perception across development are a barrier to understanding the development of audiovisual speech processing.

Some measures compatible with testing infants have been used to assess audiovisual perceptual development across the lifespan. For example, the range of asynchronies over which we bind auditory and visual speech has been estimated at various ages and shown to decrease with age [46,47,48]. Additionally, the McGurk effect—often considered a measure of audiovisual speech integration—is observed more consistently in older listeners than in infants and children [49,50]. However, it is unclear whether these measures of asynchronous and incongruent speech processing relate to the ability to benefit from naturally synchronous, congruent audiovisual speech cues in real-world, noisy backgrounds.

Recently, Lalonde and colleagues [51,52] adapted an audiovisual benefit measure from the adult literature for use with infants and children. Specifically, these studies measured audiovisual speech detection benefit by comparing detection performance in auditory-only and audiovisual conditions. In typical audiovisual speech detection experiments, adults are asked to repeatedly indicate which of two noise intervals contains acoustic speech, e.g., [26,30]. In each modality, participants are tested adaptively, decreasing the signal-to-noise ratio (SNR) after correct responses and increasing the SNR after incorrect responses, to find the SNR corresponding to a particular level of accuracy. In the auditory-only condition, no visual signal or a static image of the talker is presented during both intervals. Crucially, in the audiovisual condition, visual speech is presented in both intervals, so adults cannot simply respond based on the visual information. Improved performance in the audiovisual condition relative to the auditory-only condition occurs if adults use the correspondence between the auditory and visual speech to determine which interval contains the auditory speech. Adults can detect speech at about a 2 dB lower SNR in audiovisual conditions than in auditory-only conditions, e.g., [26,30]. Lalonde and McCreery [52] recently used this traditional method to examine development of audiovisual syllable detection benefit from school-age to adulthood and observed the same degree of benefit for 6- to 12-year-old children as for adults (about 2 dB).

**Figure 1:**
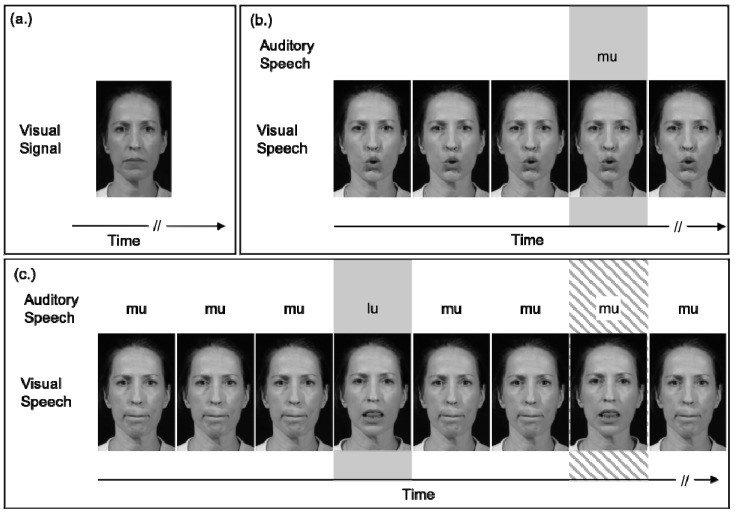
Example background, signal, and foil trial stimuli in Lalonde and Werner’s detection and discrimination tasks [51]. (**a**) Example of the visual signal for the auditory-only condition of both tasks. This single image remained on the screen throughout auditory-only testing. The auditory speech signal was identical to the audiovisual conditions. (**b**) Example of the audiovisual detection condition. The white portions of the timeline represent the background and no-signal trials. The gray portion of the timeline represents a signal trial. The visual speech repeated continuously, but auditory speech only occurred on signal trials. (**c**) Example of the audiovisual discrimination condition described in Section 6.1.2. The white portions of the timeline represent the background and no-signal trials. The gray portion of the timeline represents a signal trial, and the striped portion of the timeline represents a foil trial. The auditory and visual speech was /mu/ repeating in the background. On signal trials, both the auditory and visual speech changed. On foil trials, only the visual speech changed. Reproduced with permission from [51].

**Figure 2:**
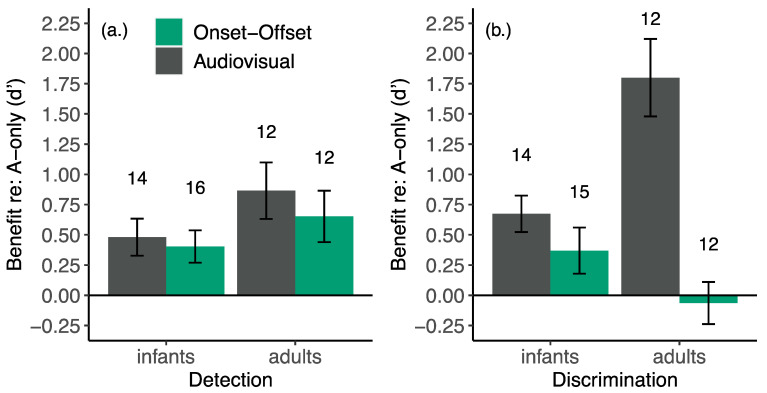
Mean and standard error of sensitivity benefit relative to the auditory-only condition for infants and adults on the (**a**) detection and (**b**) discrimination tasks. The numbers above each bar indicate the number of participants included in the condition. The visual signal in the audiovisual condition consisted of synchronous, congruent videos of the talker. In the onset-offset condition, the videos were replaced with two images of the talker, an open-mouth image that was displayed for the duration of the syllable and a closed-mouth image that was presented at all other times. The onset-offset video cued the potential timing of the acoustic syllable, but not its identity. Significant benefit was observed in all cases except adults’ discrimination in the onset-offset condition. Audiovisual benefit was significantly greater in adults than in infants on the discrimination task but not the detection task. Benefit only differed significantly across audiovisual and onset-offset conditions for adults’ discrimination. The onset-offset condition and discrimination task are discussed in greater detail in Section 6.1.2. Modified with permission from [51].

Lalonde and Werner [51] also adapted the audiovisual speech detection task for use with 6- to 8-month-old infants. Using an observer-based psychophysical procedure [53], infants and adults were trained to respond when they heard an auditory syllable /mu/ presented at random intervals in a continuous noise. The authors compared participants’ sensitivity to the auditory syllable across auditory-only and audiovisual conditions. In the auditory-only condition, infants saw a static, neutral image of the talker throughout the experiment (Figure 1a). In the audiovisual condition, a video of the talker repeating the syllable played repeatedly, even when the acoustic syllable was not presented (Figure 1b). This repeating video is the crucial difference between the current study and previous studies that have demonstrated more robust dishabituation to synchronous audiovisual speech than the isolated auditory and visual components, e.g., [18]. Because the visual speech signal played repeatedly in the background, infants could not respond in the audiovisual condition solely based on the visual information. Thus, any difference between auditory-only and audiovisual performance was because participants used the correspondence between the auditory and visual information to help determine when acoustic speech occurred. Infants and adults detected the syllable better (higher sensitivity, as measured from target detection rate and false alarm rates) in the audiovisual condition than in the auditory-only condition, suggesting that they used the correspondence between the auditory and visual information to help determine when a target trial occurred (Figure 2a). Although adults, on average, benefitted more than infants, this group difference was not statistically significant.

Overall, these results suggest that there is little change over development (from 6 months to about 30 years) in audiovisual benefit to speech detection, at least for simple speech signals such as single syllables [51]. This early ability contrasts with the protracted development of audiovisual benefit to speech recognition in children and adolescents, e.g., [34,35]. These divergent developmental trajectories underscore the need to consider what mechanisms are required to benefit on a particular audiovisual speech task and how differences in mechanisms required by different tasks and employed at different ages contribute to our understanding of audiovisual speech perception development. We believe an understanding of the development of the mechanisms that underlie audiovisual benefit will provide a more cohesive account of audiovisual speech perception development. The remainder of this paper will review mechanisms underlying audiovisual benefit and the existing research regarding the development of each mechanism. By necessity, this is not an exhaustive review of all studies related to the development of audiovisual speech perception.

## 5. Mechanisms of Audiovisual Speech Perception Benefit: Temporal and Phonetic

Audiovisual speech perception involves a large network of subcortical and cortical structures. Midbrain structures such as the superior colliculus receive both auditory and visual input, e.g., [54]. At the level of the cortex, there are direct connections between the primary sensory cortices, forward projections from primary sensory cortices to higher-level multimodal association areas such as the superior temporal sulcus (STS) and parietal, premotor, and prefrontal regions, and feedback from higher-level association areas to primary sensory cortices [55,56,57,58,59,60,61].

Neurophysiological and behavioral evidence indicates that this large neural network subserves multiple mechanisms of audiovisual speech perception benefit in adults [62,63,64,65]. More specifically, in both the adult and developmental literatures, an important distinction has been made between use of phonetic information and use of other salient auditory–visual correspondences in speech, namely temporal cues [27,43,45,51,52,62,63,64,65,66]. Terminology varies across studies, but a similar distinction has been drawn between (1) general perceptual mechanisms related to auditory–visual temporal correspondences and (2) speech-specific mechanisms that constrain phonetic and lexical interpretation.

### 5.1. General Perceptual, Temporal Mechanisms

There are correlations between the temporal properties of visible articulations and acoustic speech, with visible mouth movements preceding acoustic speech by 100 to 300 ms [20]. This means visible speech can be used to help predict the timing of acoustic speech events [26,30,65,67,68].

There are many cross-modal temporal cues in speech, varying in complexity. The most basic is the onset of speech. Onset cues increase temporal expectancy, reducing uncertainty as to when auditory speech will occur. In general, temporal expectancy makes it easier to detect sensory information. For example, defining an observation interval with a light improves detection of tones in noise compared to when the observation interval is not defined [69]. As previously noted, temporal expectancy benefits apply to speech as well [26,30,67,68]. Similar benefits are observed for speech and non-speech stimuli [70]. Simple sensitivity to auditory–visual temporal coincidence is observed in subcortical structures, including the superior colliculus [54]. Additionally, the amplitude of the auditory N1 component of electrophysiological responses is reduced when visual speech precedes the auditory signal by a small interval, a general perceptual effect related to temporal cueing of acoustic speech onset [71,72,73,74].

Beyond basic cues to speech onset/offset, there are ongoing cross-modal correlations between the amplitude envelopes of fluent auditory and visual speech signals [20,21,22]. In particular, the area of the mouth opening is correlated over time with the amplitude envelope of speech, particularly in the spectral region of the second and third formants of speech. Larger mouth openings co-occur with moments of greater speech intensity in these spectral regions.

Both mouth movements and the amplitude envelope of acoustic speech are quasi-rhythmic and temporally modulated at a rate of 2 to 7 Hz, corresponding to the timing of syllables [20]. This modulation rate is well-matched to the neural circuits involved in speech processing. Oscillations in auditory cortex at this frequency range phase lock to the amplitude envelope of the acoustic speech signal [75,76]. Phase locking to the acoustic amplitude envelope is more consistent when matched visual speech input is presented, suggesting that visual speech increases the precision of envelope tracking in auditory cortex via subcortical pathways and/or connections between cortical sensory areas [77,78]. Recent models of audiovisual speech intelligibility have embraced this idea and suggest that visual speech can be used to correct for distortions in amplitude envelope representations (e.g., due to noise or hearing loss) at the output of auditory filters in the auditory periphery [79].

Behavioral support for the use of ongoing cross-modal temporal correlations for audiovisual benefit comes from comparisons of degree of audiovisual benefit across sentences naturally varying in degree of cross-modal correlation. Grant and Seitz [26] demonstrated that audiovisual benefit to speech detection is greater for a sentence with a high cross-modal correlation than a sentence with a low cross-modal correlation, a finding that has been replicated in other studies [67,80,81]. At very difficult noise levels, this benefit extends to audiovisual speech recognition: the degree of audiovisual benefit to sentence repetition accuracy is correlated with degree of cross-modal correlation, especially in the spectral regions of the second and third speech formants [82].

Recent work has also demonstrated audiovisual benefit to sentence recognition from a visual analog of the acoustic speech amplitude envelope. When presented sentences in multi-talker babble, adults recognized speech with 3–5% greater accuracy with this visual analog (a sphere whose size correlated over time with the amplitude of the target speech signal) than when listening to auditory-only speech [83]. No significant benefit was observed for the visual analog of the acoustic amplitude envelope of a mismatched sentence. Benefit from this visual analog of the amplitude envelope is small in comparison to benefit typically observed for full visual speech signals in other studies, suggesting that adults rely on other cues from visual speech in addition to the cross-modal envelope cues.

### 5.2. Speech-Specific, Phonetic/Lexical Mechanisms

From years of experience with speech and language, adults have learned which salient visual cues are associated with phonemes, syllables, and words in their native language. For example, adults learn that a lip closure represents a bilabial sound, such as /p/, /b/, and /m/, and not a velar sound, such as /k/ or /g/. Adults use this knowledge to differentiate between visually distinct speech sounds [84]. In noisy conditions, they use visual speech to constrain interpretation to the alternatives that are consistent with the visual phonetic information [85,86]. The combination of auditory and visual cues related to speech gestures is said to involve higher-level association areas such as the STS, which may play a role in weighting of auditory and visual information by modulating functional connectivity with primary sensory regions [65,87]. The use of visual phonetic knowledge is observable from differences in the patterns of consonant confusion errors in auditory-only and audiovisual word recognition in noise. Although error rates are lower in audiovisual conditions than in auditory-only conditions, adults are three times more likely to substitute a consonant with a visually similar one (e.g., substituting for a consonant with the same place of articulation) when making an error in audiovisual conditions than when making an error in auditory-only conditions [85].

This concept extends to the domain of lexical activation [86]. The neighborhood activation model of speech perception suggests that when we hear a word, a neighborhood of phonetically similar words is activated [88]. Words with small/sparse neighborhoods are more easily recognized in noise than words with large/dense neighborhoods, because there are fewer words competing for lexical selection [88]. Mattys et al. [89] demonstrated that visual equivalents of lexical neighborhoods are activated during speechreading. In audiovisual conditions, auditory neighborhood density, visual neighborhood density, and the density of the overlapping auditory and visual lexical neighborhoods all contribute to recognition of audiovisual words [86]. This suggests that visual speech constrains lexical selection during audiovisual speech perception.

Further support for the use of visual speech to activate lexical representations comes from the word superiority effect in audiovisual speech perception [90]. Fort et al. [90] asked adults to listen for the presence of a particular phoneme in a series of words and non-words presented in noise. Using a go/no-go task, they were told to press a button as soon as they heard the target phoneme. Participants detected the phoneme faster and with greater accuracy when targets were presented audiovisually than when they were presented auditorily. A word superiority effect was observed, with greater accuracy of phoneme detection for words than for non-words, but only in the audiovisual condition. This result was taken as evidence that visual phonetic information contributes to lexical activation during word recognition.

## 6. Development of the Mechanisms of Audiovisual Speech Perception Benefit

The temporal and phonetic mechanisms of audiovisual benefit provide a framework from which to interpret studies relevant to development of audiovisual speech benefit. From a neurophysiological perspective, the cortical structures used to access visual/multimodal phonetic/lexical representations are experience-based and show limited maturation during the first year of life. In contrast, the subcortical pathways that underlie sensitivity to auditory and visual synchrony are highly developed at 6 months of age [91,92].

In addition to distinguishing between temporal and phonetic cues, one has to consider the complexity and salience of the particular temporal or phonetic cues needed to perform a task. More basic auditory–visual temporal cues, such as onset cues, and more visually distinct or visually salient phonetic cues appear to contribute to audiovisual speech perception benefit earlier in development than more subtle and complex cues. Studies of general auditory–visual temporal perception in infancy indicate that sensitivity to synchronous onsets emerges first, with sensitivity to increasingly complex temporal cues (duration, rate, and rhythm information) emerging sequentially and in a hierarchical fashion [93]. Similarly, recent studies of auditory–visual vowel mapping show that whether infants demonstrate a preference for matched vs. mismatched vowels depends on the distinctiveness of the vowel contrast [94] and on the distinctiveness of the particular talker’s visual articulations [95].

The importance of visual cue salience/distinctiveness extends to later development. One-year-old infants recognize mispronunciations in familiar audiovisual words if the mispronunciation involves a change in a visually salient feature (place) but not in a visually indistinguishable feature (voicing) [96]. Three- and four-year-olds benefit from visual speech when discriminating consonants that are visually distinct to adults (ba vs. ga), but not consonants that are less visually distinct to adults (ba vs. ma) [85]. Adults benefit from visual speech for both contrasts, but the audiovisual advantage is stronger for the visually salient contrast, suggesting visual cue salience is similar for preschoolers and adults, but the ability to benefit from less salient visual cues is late developing [85]. Finally, whereas 4- to 15-year-old children can all use visual speech to fill-in an acoustically missing word-initial /b/ (which is visually salient due to bilabial place of articulation), they do not do so for a missing /g/ (which is less visually salient due to velar place of articulation) until age 8 years [97].

### 6.1. Distinguishing Use of Temporal and Phonetic Cues in Development

A few developmental studies have been designed to differentiate between audiovisual speech perception benefits resulting from the use of temporal and phonetic cues, respectively. One approach is to degrade the acoustic signal in ways that minimize the use of phonetic cues and compare performance with and without those phonetic cues. A second approach is to limit the phonetic cues provided by the visual signal and compare benefit from full and cue-limited visual signals. A third approach is to vary the level of perceptual processing required by a task, from speech detection benefit (which we have noted is based on temporal cues in adults) to higher-level tasks such as speech discrimination and speech recognition (which likely rely on phonetic cues). Fourth, by examining the patterns of errors in auditory and audiovisual word recognition, we can assess whether visual phonetic information was used for audiovisual benefit. Finally, different components of electrophysiological responses to audiovisual speech stimuli may vary in their dependence on temporal and phonetic cues.

#### 6.1.1. Distinguishing Use of Temporal and Phonetic Cues in Development with Sine Wave Speech

Baart and colleagues [43,66] have examined whether infants’, children’s, and adults’ ability to match tri-syllabic non-words across modalities is based on temporal or phonetic cues by using sine wave speech (SWS). SWS is an acoustic speech signal that has been reduced to three sinusoids representing temporal variation in the first three formants of speech [98]. SWS preserves the temporal characteristics of speech and the amplitude of the formants that correlate with the visual amplitude envelope [20,26], but other phonetic information is severely degraded. Decrements in performance with SWS in comparison to unprocessed speech are believed to reflect the removal of phonetic cues; Any remaining ability with SWS is said to have resulted from reliance on temporal cues [43].

Baart and colleagues [43] compared infants’ and adults’ ability to match trisyllabic non-words across auditory and visual domains with unprocessed speech and with SWS. Adults matched unprocessed speech to visual speech better than they matched SWS to visual speech, suggesting that they relied—to some degree—on phonetic information. In contrast, infants’ preference for matching visual speech was similar for unprocessed speech and SWS, suggesting that they relied on the temporal characteristics that were preserved in SWS. The authors argued that infants do not need phonetic information to detect the auditory–visual correspondence when there are salient non-phonetic (i.e., temporal) cues.

Naïve observers are typically unaware that SWS is generated from a speech signal, but once informed of its speech-like nature, they can usually understand it [98]. Therefore, differences between performance of naïve and informed observers are believed to reflect non-speech and speech processing modes, respectively. When identical performance is observed between naïve and informed observers on an audiovisual speech task, it is said that they relied on temporal cues. This is the case for audiovisual speech detection benefit and judgments of temporal order and synchrony [62,99]. If an effect only occurs with observers who are aware of the speech-like nature of the signals, as with the McGurk effect, the effect is said to be speech-specific [62,99,100,101].

In a follow-up to the infant experiment, Baart and colleagues [66] compared 4- to 11-year-old children’s matching ability when naïve to the speech-like nature of SWS to that after being trained to recognize the SWS as speech. Adult pilot subjects matched the SWS to the visual speech better after training. Children 4 to 6.5 years of age appeared to rely on temporal cues and matched the auditory and visual speech equally well before and after training. However, after 6.5 years, children matched better after training, suggesting that they begin to use phonetic cues around that age. The authors argued that this represents the point in development at which phonetic processing is sufficiently mature to influence audiovisual speech matching.

#### 6.1.2. Distinguishing Use of Temporal and Phonetic Cues in Development: Limiting Visual Cues

In previous sections we noted that (1) infants, children, and adults demonstrate equal audiovisual speech detection benefit for syllables [51,52], and (2) detection benefits result from the use of simple cues that increase temporal expectancy, e.g., [30]. This suggests that the use of basic temporal cues to speech onset develops early. In fact, Lalonde and Werner [51] demonstrated that the use of visual cues to the onset and offset of visual speech could account for detection benefit in both infants and adults. In addition to auditory-only and audiovisual conditions, Lalonde and Werner’s [51] audiovisual detection experiment included a third condition with a visual signal that only cued the onset and offset of visual speech and eliminated all other potential visual cues from the talker. In this condition, the videos of the talker saying /mu/ were replaced with two pictures of the talker: an open-mouthed picture that was presented for the duration of the syllable, and a closed-mouth picture that was presented at all other times. Both infants and adults benefited from this onset/offset cue. Benefit from the onset/offset cue was not significantly different than benefit from the full audiovisual speech signal (Figure 2a), suggesting that the use of onset cues to increase temporal expectancy accounts for syllable detection benefit in both infants and adults.

There is an assumption that cues that benefit speech detection also apply to higher-level tasks such as speech recognition, due to the hierarchical nature of speech perception. One must detect speech in order to discriminate its features and recognize phonemes and words. To test this assumption, Lalonde and Werner [51] conducted a second experiment, examining audiovisual benefit to the slightly higher-level task of speech discrimination. Procedures were similar to the detection task, with a few exceptions. Infants and adults heard the syllable /mu/ play repeatedly in continuous noise and were trained to respond when they heard a different syllable (/gu/ or /lu/) (Figure 1c). In the auditory-only condition, participants saw a neutral image of the talker throughout the experiment. In the onset/offset condition, the videos were once again replaced with images of the talker that only cued the onset and offset of syllables. In the audiovisual condition, synchronous and congruent videos played during the background syllables and during the target trials. To ensure participants could not respond solely based on visual information, the audiovisual condition also included incongruent foil trials, during which the visual signal changed, and the auditory signal did not (visual /gu/ or /lu/ with auditory /mu/). Participants were trained not to respond to these foil trials. Infants and adults discriminated audiovisual syllables in noise better than auditory-only syllables (Figure 2b). However, in this case, infants benefited far less than adults. Further, adults benefited much more from the full audiovisual signal than the onset/offset cue, suggesting that they relied on other salient differences between the visual syllables (rather than onset/offset cues) for discrimination benefit. Infants benefitted equally from the onset/offset cue and full audiovisual cue for discrimination, suggesting that they continued to rely on synchronous onsets and offsets even when more sophisticated cues were available. Results from these two experiments suggest that infants are rather mature in their ability to use onset cues to benefit both speech detection and speech sound discrimination but are still developing in their use of other salient differences and/or phonetic cues.

Infants can use more complex cross-modal temporal cues in fluent speech for audiovisual benefit. Hollich and colleagues [102] used a head-turn preference procedure to examine whether 7.5-month-old infants could use synchronous visual speech to segregate competing auditory speech streams, attend to the “target” stream, and segment common target words from the passage. Groups of infants listened to a story read by a female talker using infant-directed speech while a competing male talker reading the methods section of a research paper played in the background at an equal intensity level. Infants were presented the story with (1) a static image of the target talker, (2) synchronous, congruent visual speech, (3) asynchronous, incongruent visual speech, or (4) a moving oscilloscope pattern which removed all visual speech cues except the temporal envelope of the speech signal.

All groups were subsequently tested in auditory-only conditions to determine whether infants showed preference for the target word spoken by the same female target talker over a non-target word spoken by the same talker in the same manner. Infants who heard the synchronous, congruent passage demonstrated preference for the familiar target word, while those who saw a static image or asynchronous, incongruent speech did not. This suggests that infants used the congruent visual cues to segregate and attend to the target speech stream and to segment the target words from the passage. Importantly, infants who viewed the oscilloscope pattern demonstrated the same preference, suggesting that infants’ ability to benefit from the visual speech signal was related to the use of correlated amplitude envelope cues. Adults’ benefit from a visual analogue of the amplitude envelope of speech is also significant, but is greatly decreased in comparison to synchronous, congruent visual speech [83]. It is impossible to say from the methods used by Hollich and colleagues [102] whether or not infants benefit more from full visual speech than the visual envelope cue.

#### 6.1.3. Distinguishing Use of Temporal and Phonetic Cues in Development: Varying Level of Perceptual Processing Required by a Task

As previously noted, adults seem to rely on different cues to benefit from visual speech depending on the level of processing required by an audiovisual speech perception task. They relied on onset/offset cues for audiovisual benefit to speech detection and relied on other salient differences and/or phonetic cues for discrimination [51]. Among school-age children and adults, studies have attempted to distinguish between use of general perceptual (temporal) mechanisms and speech-specific (phonetic) mechanisms by examining developmental differences in audiovisual benefit across tasks varying in the level of perceptual processing required: a detection task that only requires basic awareness of speech, a discrimination task that requires perceiving a salient difference between background and target speech utterances, and a recognition task that requires recognizing that exemplars belong to the same lexical category despite variable sensory input [27]. The stimuli, experimental paradigm, SNR, and chance level of performance was the same for all tasks. Children demonstrated the same benefit as adults on the detection and discrimination tasks, but less benefit than adults on the recognition task. One interpretation of this result is that, whereas audiovisual benefit to detection and discrimination results from general perceptual mechanisms, such as sensitivity to temporal correspondences, the additional audiovisual benefit to recognition in adults results from accessing visual/multimodal phonetic categories. Thus, 6- to 8-year-old children were adult-like in their use of general perceptual mechanisms but still developing in their use of phonetic mechanisms.

#### 6.1.4. Distinguishing Use of Temporal and Phonetic Cues in Development: Examining Errors for Patterns Consistent with Use of Visual Phonetic Cues

Lalonde and Holt [85] examined whether preschoolers use phonetic mechanisms for audiovisual benefit to word recognition in noise. They examined patterns of consonant confusion errors in auditory-only and audiovisual word recognition in noise to determine whether 3- and 4-year-old children used visual speech to constrain phonetic interpretation to alternatives that are consistent with visual phonetic information. Children completed a word recognition-in-noise task in auditory-only and audiovisual conditions. Preschoolers benefitted significantly from the presence of visual speech, but to a lesser extent than adults (13% vs. 42% respectively). Patterns of consonant confusion errors were compared across auditory-only and audiovisual conditions. Each consonant substitution was categorized according to whether the consonant was substituted for a visually similar consonant or for a consonant that adults should be able to tell apart visually.

Adults were three times more likely to substitute a consonant with a visually similar one in audiovisual conditions than in auditory-only conditions. Four-year-olds’ errors followed a similar pattern, with a 2:1 difference between audiovisual and auditory-only substitutions. Three-year-olds, however, showed no significant difference in visually salient substitution errors for auditory-only compared to audiovisual conditions. This suggests that by 4 years of age children may have reached a point in development at which visual phonetic processing is mature enough to benefit audiovisual word recognition in noise. The same 3-years-old children are sensitive enough to salient differences between visual consonants that they can use them for discrimination benefit (see Section 6), but they do not seem to use this sensitivity in the process of audiovisual word recognition in noise [85]. This developmental time course contrasts with cross-modal matching results that suggested that children may not use visual phonetic cues for cross-modal matching until 6.5 years of age [66].

#### 6.1.5. Distinguishing Use of Temporal and Phonetic Cues in Development: Neural Responses

Audiovisual effects on some early-stage components of electrophysiological responses, such as the auditory N1, are related to visual speech’s ability to temporally cue the onset of acoustic speech [71,72,73,74]. Other early-stage components, such as P2, seem to reflect audiovisual phonetic binding [103]. For example, using SWS stimuli, Baart et al. [103] demonstrated that differences in the N1 in response to audiovisual conditions, relative to unimodal conditions, occur both for observers who are aware of the speech-like nature of SWS and those who are not informed. In contrast, differences in the P2 component only occur for observers who are aware of the speech-like nature of SWS. This suggests that age-related differences in the effects of visual speech on N1 and P2 may reflect the use of temporal and phonetic cues, respectively.

Kaganovich et al. [104] examined electrophysiological responses to auditory, visual, and audiovisual syllables in 7- to 8- and 10- to 11-year-old children and adults. Although morphology of the electrophysiological responses differed across age groups, differences in responses to audiovisual stimulation—relative to the combined responses to unimodal auditory plus unimodal visual stimulation—were similar across children and adults, for both the N1 and P2 components. These findings were interpreted as suggesting that early-stage audiovisual speech processing effects are mature by at least 7 years of age. Although these early-stage components are adult-like, the complex neural networks that contribute to audiovisual speech perception include structures that vary in their developmental trajectory, with some structures developing into adolescence [105]. Additionally, there are differences between adults and 8- to 11-year-old children in the functional interactions among these various regions [106]. Development of the neurophysiological mechanisms underlying audiovisual benefit in human infants and children remains an understudied area.

### 6.2. Development of the Use of Phonetic Cues for Audiovisual Speech Perception Benefit

Evidence suggests that infants are sensitive to audiovisual phonetic cues [36,37,38,39,40,41,96,107,108]. Infants show preference for a face articulating the vowel they are hearing over a vowel that does not match, even when there are no temporal cues to distinguish between the two [36,37,38,39,40,41]. They are also sensitive to audiovisual phonetic mismatches [63]. However, the fact that infants’ audiovisual benefit is not greater for combined temporal and phonetic cues than for temporal cues alone [51] suggests that phonetic cues may be less important for infants’ audiovisual benefit. Additional studies are needed to directly address this open question.

Like infants, 3- and 4-year-olds are sensitive to the correspondence between auditory and visual speech cues. They can match acoustic vowels to the correct facial articulations with greater than chance accuracy in the absence of any temporal cues to distinguish between the two [85]. Adults [109], 12-month-olds [96], and preschoolers [85] are all sensitive to visual cue salience, in that they demonstrate greater sensitivity to visual differences in place of articulation than visual differences in manner of articulation or voicing. However, the ability to use visual speech during word recognition to constrain phonetic interpretation does not appear to emerge until between 3 and 4 years of age [85]. Further, development of the use of speech-specific mechanisms of audiovisual benefit extends at least past age 8 years [27] and likely into adolescence when audiovisual benefit to speech recognition in noise reaches maturation [34,35].

### 6.3. Development of the Use of Lexical, Semantic, and Syntactic Cues for Audiovisual Speech Perception Benefit

Differentiating between the use of phonetic and lexical cues for audiovisual benefit is perhaps even more difficult than differentiating between phonetic and temporal cues. Nevertheless, a couple of studies have been completed that speak to the use of lexical cues. To test children’s use of visual speech to activate lexical representations, Fort et al. [110] tested for a word superiority effect in audiovisual speech. Recall that in audiovisual conditions, adults can perform a phoneme detection task with greater accuracy for words than for non-words [90]. This result was taken as evidence that visual phonetic information contributes to lexical activation during word recognition. Fort et al. [110] conducted a similar study with children, age 5 to 10 years. To make the task easier, children monitored for particular target vowels—rather than consonants—in words and pseudowords. Beginning in first grade (age 6–7 years), children showed a clear audiovisual advantage for vowel monitoring, with greater vowel detection accuracy in audiovisual than auditory-only conditions and a significant overall advantage for words over nonwords. However, the word superiority effect did not differ across modalities for any age group. This suggests that whereas visual speech contributes to lexical activation in adults, it may contribute to phonetic, but not lexical, processing in children up to 10 years of age. Recent electrophysiological results corroborate this finding and suggest that children may not use visual speech for lexical access until sometime after age 12 years [111]. Most studies of audiovisual benefit development have relied on isolated words and syllable stimuli. Therefore, the use of sentence-level syntactic and semantic processing in audiovisual benefit remains an understudied area.

## 7. Conclusions

Sensitivity to the correspondence between auditory and visual speech cues is apparent shortly after birth [44] and is observed throughout the first year of life [36,37,38,39,40,41]. This early sensitivity contrasts with protracted development—into adolescence—in the ability to use visual speech to compensate for the noisy nature of our everyday world [34,35]. Inconsistencies in the literature regarding the development of audiovisual speech perception likely reflect three facts.

First, infants and children often use different mechanisms than adults to benefit from visual speech. An important distinction—supported by both behavioral and neurophysiological evidence—has been drawn between general perceptual audiovisual benefit resulting from the use of temporal cues and speech-specific audiovisual benefit resulting from the application of visual/multimodal phonetic knowledge. The use of temporal cues for general perceptual benefit emerges early and does not improve with development. At 6 months of age, infants can use temporal cues to the onset of speech to better detect and discriminate syllables in noise [51]. Adults also rely on temporal cues for speech detection, and benefit to speech detection does not vary between infants, children, and adults [51,52]. At 7 months of age, infants can also use a visual analogue of the acoustic speech amplitude envelope to segregate competing auditory speech streams, attend to the “target” stream, and segment common target words from a passage [102]. In cases where adults rely on more sophisticated cues for audiovisual benefit or auditory–visual speech matching, infants continued to rely on synchronous onsets and offsets [51] and other temporal cues [43]. It is unclear when in development the use of phonetic cues begins. Studies evaluating audiovisual speech benefit in infancy unequivocally suggest that benefit results from temporal cues, but it is possible that methods appropriate for testing audiovisual speech benefit in infants are better aligned with the use of temporal cues. Error patterns on audiovisual speech recognition tasks suggest that visual phonetic cues are first used for word recognition benefit at age 4 years [85], whereas phonetic cues may not be used for cross-modal speech matching until age 6.5 years [66]. Visual speech cues may not be used for lexical access until adolescence [110,111].

Second, different testing methods target different underlying audiovisual mechanisms. Infant matching studies measure whether infants are sensitive to auditory-visual correspondences, whereas child studies measure whether children can use this correspondence to benefit from visual speech cues. Techniques compatible with testing infants typically cannot differentiate between sensitivity to the correspondence between auditory and visual speech cues and use of these correspondences for audiovisual benefit. Further, the level of perceptual processing required by a task and its emphasis on phonetic processing and lexical access affect the types of cues that are used at any given stage of development [27,51,110].

Finally, the complexity and salience of the particular temporal or phonetic cues needed to perform an audiovisual speech task affect whether an infant or child can use it. Development of general sensitivity to audiovisual temporal cues during the first year of life is sequential and hierarchical; sensitivity to onset cues develops first and bootstraps later development of sensitivity to more complex temporal cues [93]. This suggests that development of the use of increasingly complex temporal cues in audiovisual speech may also proceed in a sequential and hierarchical fashion. Additionally, the physical distinctiveness of particular visual speech sound contrasts affects both sensitivity to audiovisual correspondences in infancy [94,95] and the ability to use visual speech in childhood and adulthood [96,97].

Given these three facts, careful experimental design is necessary to determine what cues are used on any particular audiovisual speech perception task. Stimulus manipulations that decrease the auditory or visual cues available to observers help to disambiguate what cues are being used, as do comparisons across tasks differing in complexity. A caveat about using these stimulus manipulations is that they decrease ecological validity. A more cohesive account of the development of audiovisual speech perception may follow from a more thorough understanding of the development of sensitivity to and use of various temporal and phonetic cues.

## Data Availability

No new data were created or analyzed for this review. Data sharing is not applicable to this article.
